# Smoking and mortality risk in COPD patients: a prospective cohort study in Sichuan, China

**DOI:** 10.3389/fmed.2026.1842930

**Published:** 2026-05-29

**Authors:** Boke Chen, Di Wu, Xiaofang Chen, Xia Wu, Mingqiang Yuan, Yujin He, Zhuo Wang, Xueli Zhang, Jun Lv, Canqing Yu, Pei Pei, Dianjianyi Sun, Xianping Wu, Xiaofang Chen

**Affiliations:** 1 Department of Epidemiology and Biostatistics, School of Public Health, Chengdu Medical College, Chengdu, China; 2 Pengzhou Disease Control and Prevention Center, Pengzhou, Sichuan, China; 3 Sichuan Center for Disease Control and Prevention, Chengdu, China; 4 Sichuan Health Information Center, Chengdu, China; 5 Department of Epidemiology & Biostatistics, School of Public Health, Peking University, Beijing, China; 6 Peking University Center for Public Health and Epidemic Preparedness and Response, Beijing, China; 7 Key Laboratory of Epidemiology of Major Diseases (Peking University), Ministry of Education, Beijing, China; 8 Sichuan Provincial Health and Health Commission, Chengdu, China; 9 Key Laboratory of Intelligent Medical Care and Elderly Health Management, Chengdu Medical College, Chengdu, Sichuan, China

**Keywords:** COPD, CVD, mortality risk, prospective study, smoking

## Abstract

**Purpose:**

To assess the association between smoking and mortality in patients with chronic obstructive pulmonary disease (COPD) in Sichuan Province, China.

**Methods:**

Data concerning COPD patients in Pengzhou, Sichuan was collated from the China Kadoorie Biobank (CKB). COPD was defined as post-bronchodilator forced expiratory volume in 1 s/forced vital capacity of <70%. The relationship between smoking and mortality was evaluated by Cox proportional hazards regression.

**Results:**

Eight thousand five hundred one COPD patients, aged 30–79 years, were enrolled enabling analysis of 85,600 person-years of follow-up data (mean follow-up: 10.07 years). Two thousand deaths occurred during the period of investigation, including 665 from COPD and 1,116 from cardiovascular disease (CVD). Ex-smokers were at higher risk of both all-cause mortality [hazard ratio (HR) 1.56; 95% confidence interval (CI) 1.36–1.80], COPD (HR 2.24; 95% CI 1.80–2.80) and CVD mortality (HR 1.54; 95% CI 1.28–1.85) than those who had never smoked. An increased duration of smoking was associated with higher risk of death. A dose–response relationship was observed. Patients who smoked > 20 g tobacco/day had 35% higher all cause, 66% higher COPD and 34% higher CVD mortality risk. Those with cumulative exposures ≥ 250,000 g had the highest risks of all outcomes. Earlier age of smoking onset and oral only smoke intake (as opposed to lung inhalation) were also linked to greater mortality risk.

**Conclusion:**

Smoking was an independent risk factor for COPD-specific, CVD and all-cause mortality in COPD patients from Sichuan with higher tobacco consumption giving a stronger association.

## 1 Introduction

Chronic obstructive pulmonary disease (COPD) is a major global public health challenge, characterized by partially reversible but persistent airflow limitation (1). Approximately 90% COPD-specific deaths occur in low- and middle-income countries and the condition is the third leading cause of worldwide mortality, according to the WHO (2). Indeed, global burden of disease (GBD) studies have attributed over 3 million deaths per annum or 6.0% total global mortality to COPD (3). The COPD mortality rate was 125.43 per 100,000 of the population in Sichuan Province in 2023 with higher mortality among adults aged ≥ 60 years (4). The primary causes of death were COPD or cardiovascular disease (CVD) (5) which were among the top three causes of single-disease mortality in Sichuan residents (6). CVD accounted for 15.8–39% of all mortality in the same population (5, 7).

Tobacco smoking is considered a significant risk factor for mortality from all causes, COPD and CVD (8) and was identified as a leading preventable cause of death worldwide by a GBD study conducted in 2019 (9). The presence of the chronic respiratory condition increases susceptibility to harmful exposure by inhalation (10) and COPD patients merit particular attention with respect to the health impact of tobacco use. Pre-existing studies have generally focused on the general population with few investigating high-risk COPD patients. Smoking status has been previously addressed in this context but inhalation depth (deep vs. shallow) or age at smoking initiation have rarely been scrutinized.

The current study accessed data from the Pengzhou site of the China Kadoorie Biobank (CKB) and examined the impact of smoking inhalation depth and age of smoking onset on mortality from COPD, CVD and all causes in a cohort of COPD patients. The aim was to define mortality risk patterns in this high-risk population to inform targeted interventions and public-health policy regarding COPD management.

## 2 Methods

### 2.1 Baseline survey

A total of 512,723 participants from 10 geographically diverse Chinese regions (5 urban and 5 rural) were recruited by the CKB between 2004 and 2008 and detailed methodology has been published previously (11–13). All participants completed baseline surveys. All experiments were completed in accordance with the Declaration of Helsinki (amended 2023) and all participants gave written informed consent for collection and publication of data. The study protocol was approved by the University of Oxford Research Ethics Committee (Ref: 025–04) and the Chinese Center for Disease Control and Prevention Ethical Review Committee (Approval: 005/2004).

Pengzhou, Sichuan is a rural area from which 55,686 participants, aged 30–79, contributed complete baseline data to the CKB. Eight thousand six hundred forty-three cases of COPD were identified by either post-bronchodilator forced expiratory volume in 1 s/forced vital capacity of < 0.70 (14) or physician-diagnosed chronic bronchitis/emphysema/cor pulmonale in township-level or higher hospitals. Patients with self-reported cancer (*n* = 125) or asthma (*n* = 223) at baseline were excluded and the remaining 8,501 COPD patients included.

### 2.2 Exposure variables

Participants were divided into four groups of those who had never smoked, occasional smokers, ex-smokers and current smokers based on questionnaire responses. Occasional smokers reported smoking fewer than 2 cigarettes per month. Current smokers smoked regularly at the time of the survey and had smoked > 100 cigarettes in their lifetime. Ex-smokers had previously smoked regularly before ceasing > 6 months prior to the survey. Smoking duration was calculated from the difference between age of smoking onset and age at the study date or at cessation giving four categories: never/occasional smokers, <30 years, 30–50 years and ≥50 years. Cigarette equivalents/day were estimated from self-reported tobacco types and amounts with conversion to a cumulative smoking baseline calculated using methods reported previously (15–17). Mean cigarette equivalents/day were categorized into never/occasional smokers, <10, 10–20 or ≥20/day. Cumulative smoking exposure at baseline was expressed as never/occasional, 1–<100,000, 100,000–<250,000 or ≥250,000 g and age at smoking initiation as never/occasional, < 15, 15–25 or ≥25 years. Tobacco types consumed by current or ex-smokers were categorized as cigarettes (filtered or unfiltered), other tobacco products (hand-rolled tobacco, pipes, water pipes or cigars) and mixed. The depth of smoke inhalation was self-reported as either oral only (smoke expelled after entering the mouth) or deep inhalation (smoke reaching the throat or lungs).

Sociodemographic characteristics of sex, age, educational level, occupation, marital status and annual household income; lifestyle factors of smoking category, secondhand smoke exposure, alcohol consumption, body mass index [BMI], physical activity level and fresh-fruit intake; as well as health-related factors including family history of CVD, hypertension and diabetes were also recorded. Secondhand smoke exposure was classified as never/occasional, 1–2 days/week, 3–5 days/week or daily/almost daily exposure to tobacco smoke from others in the home, workplace or public places for >5 min. Alcohol consumption was categorized as yes (regular/occasional/weekly/monthly/former drinking) or no (never). BMI (kg/m^2^) was classified as underweight (<18.5), normal (18.5–<24.0), overweight (24.0–<28.0) or obese (≥28.0). The daily physical activity level was calculated in metabolic equivalent [MET]-h/day, as described previously (18), and grouped into tertiles of low (<16), medium (16–26) or high (>26). Fresh-fruit intake was categorized as frequent (4–7 days/week) or occasional (0–3 days/week). A family history of CVD, hypertension, and diabetes were all self-reported and categorized as binary variables (yes or no). Cough and morning phlegm over the previous 12 months is expressed as yes (symptoms present) or no (no symptoms).

### 2.3 Mortality ascertainment

Disease outcomes were coded according to the International Classification of Diseases, Tenth Revision (ICD-10). Primary endpoints were mortality due to all causes, COPD (ICD-10: J41–J44) or CVD (ICD-10: I00–I99). Follow-up duration ran from baseline survey date to date of initial disease onset, date of loss to follow-up or study end date (December 31, 2017). Mortality and morbidity data were collated from the national Disease Surveillance Points system, Death Cause Registry and Universal Medical Insurance databases and supplemented by community visits, household surveys and telephone follow-ups. The CKB project had a low loss-to-follow-up rate of 0.83% as of 2021 (19).

### 2.4 Statistical analysis

Data cleaning and analysis were performed with SAS 9.4 and forest plots were generated by R 4.3.3. Categorical variables are presented as n (%) and inter-group comparisons were made by *χ^2^* tests. Associations between smoking category and covariates, mortality from all causes, COPD and CVD were analyzed using multivariate Cox proportional hazards regression models adjusted for potential confounders, including sociodemographic factors, lifestyle factors, body mass index (BMI), physical activity, fresh fruit intake, secondhand smoke exposure, and family history of cardiovascular disease. Subgroup analyses were stratified by sex and age. Sensitivity analyses were performed to assess the robustness of the findings, including exclusion of participants with respiratory symptoms, early deaths within 2 years of follow-up, and extreme values of cumulative smoking exposure (≥99th percentile). All statistical tests were two-sided, and a *p*-value < 0.05 was considered statistically significant.

## 3 Results

### 3.1 Descriptive statistics

Eight thousand five hundred one COPD patients, 43.10% male and 56.90% female, had a mean age of 57.64 ± 10.41 years. There was a significant sex disparity among the 56.73% of the cohort who had a history of smoking with 77.32% males being current smokers and 89.10% females having never smoked. Most patients were aged ≥ 50 years with 33.29% being 50–59 years, 84.41% were married, 88.37% engaged in agricultural occupations, 79.44% had only a primary education or less and 71.44% had a low household income of <10,000 Chinese yuan per annum. 62.08% patients had a BMI in the normal range of 18.5–23.9 kg/m^2^, 54.75% reported low physical activity levels, 83.30% occasional fruit intake and 36.56% no alcohol consumption. A family history of CVD was reported by 13.13% of participants, while 7.16% had hypertension and 1.76% had diabetes (Table 1).

**Table 1:** Baseline characteristics [*n* (%)].

Characteristics	Overall	Smoking category			
		Never smoked	Occasional smoker	Ex-smoker	Current smoker
Sex					
Men	3,664(43.10)	401(10.90)	282(39.89)	523(55.82)	2,458(77.32)
Women	4,837(56.90)	3,277(89.10)	425(60.11)	414(44.18)	721(22.68)
Age (years)					
30-	636(7.48)	463(12.59)	37(5.23)	17(1.81)	119(3.74)
40-	1,233(14.50)	748(20.34)	75(10.61)	35(3.74)	375(11.80)
50-	2,830(33.29)	1,310(35.62)	242(34.23)	247(26.36)	1,031(32.43)
60-	2,712(31.90)	862(23.44)	243(34.37)	419(44.72)	1,188(37.37)
70–79	1,090(12.82)	295(8.02)	110(15.56)	219(23.37)	466(14.66)
Marital status					
Never married	88(1.04)	18(0.49)	11(1.56)	4(0.43)	55(1.73)
Married	7,176(84.41)	3,177(86.38)	571(80.76)	748(79.83)	2,680(84.30)
Sep/Div/Wid	1,237(14.55)	483(13.13)	125(17.68)	185(19.74)	444(13.97)
Occupation					
Factory workers	76(0.89)	33(0.90)	5(0.71)	8(0.85)	30(0.94)
Agricultural	7,512(88.37)	3,240(88.09)	648(91.65)	767(81.86)	2,857(89.87)
Other	913(10.74)	405(11.01)	54(7.64)	162(17.29)	292(9.19)
Education level					
No formal school	2,201(25.89)	930(25.29)	212(29.99)	311(33.19)	748(23.53)
Primary school	4,552(53.55)	1807(49.13)	373(52.76)	516(55.07)	1856(58.38)
Junior high school or above	1748(20.56)	941(25.58)	122(17.26)	110(11.74)	575(18.09)
Annual household income (yuan)					
<10,000	6,073(71.44)	2,578(70.09)	524(74.12)	685(73.11)	2,286(71.91)
10,000–20,000	1926(22.66)	848(23.06)	151(21.36)	205(21.88)	722(22.71)
≥20,000	502(5.91)	252(6.85)	32(4.53)	47(5.02)	171(5.38)
BMI (kg/m^2^)					
<18.5	916(10.78)	316(8.59)	69(9.76)	132(14.09)	399(12.55)
18.5–24.0	5,277(62.08)	2,135(58.05)	420(59.41)	540(57.63)	2,182(68.64)
24.0–28.0	1818(21.39)	957(26.02)	168(23.76)	198(21.13)	495(15.57)
>28.0	490(5.76)	270(7.34)	50(7.07)	67(7.15)	103(3.24)
Physical activity level					
Low	4,654(54.75)	1819(49.46)	395(55.87)	655(69.90)	1785(56.15)
Moderate	3,287(38.67)	1,608(43.72)	272(38.47)	244(26.04)	1,163(36.58)
High	560(6.59)	251(6.82)	40(5.66)	38(4.06)	231(7.27)
Fresh fruit intake frequency					
Regular	1,420(16.70)	761(20.69)	101(14.41)	165(17.61)	390(12.27)
Occasional	7,081(83.30)	2,917(79.31)	603(85.29)	772(82.39)	2,789(87.73)
Alcohol consumption status					
Never regular	3,108(36.56)	1986(54.00)	192(27.16)	276(29.46)	654(20.57)
Ex-regular	565(6.65)	93(2.53)	46(6.51)	184(19.64)	242(7.61)
Occasional	2,295(27.00)	1,266(34.42)	247(34.94)	188(20.06)	594(18.68)
Monthly	143(1.68)	43(1.17)	25(3.54)	10(1.07)	65(2.04)
Reduced intake	495(5.82)	72(1.96)	55(7.78)	108(11.53)	260(8.18)
Weekly	1895(22.29)	218(5.93)	142(20.08)	171(18.25)	1,364(42.91)
Family history of cardiovascular disease					
No	7,385(86.87)	3,177(86.38)	621(87.84)	811(86.55)	2,776(87.32)
Yes	1,116(13.13)	501(13.62)	86(12.16)	126(13.45)	403(12.68)
Hypertension					
No	7,854(92.39)	3,398(92.39)	653(92.36)	830(88.58)	2,973(93.52)
Yes	647(7.61)	280(7.61)	54(7.64)	107(11.42)	206(6.48)
Diabetes					
No	8,351(98.24)	3,615(98.29)	691(97.74)	914(97.55)	3,131(98.49)
Yes	150(1.76)	63(1.71)	16(2.26)	23(2.45)	48(1.51)

A cumulative 85,612.30 person-years of follow-up (mean: 10.07 years) and 2,000 deaths (23.53% mortality), including 665 from COPD (7.82%) and 1,116 from CVD (13.13%), had occurred by December 31, 2017. Stratified analyses showed elevated all cause, COPD and CVD mortality rates among males, unmarried or non-cohabitating individuals (separated/divorced/widowed or never married), those with “other” occupational classifications, underweight patients, infrequent fresh fruit consumers, former regular alcohol drinkers and those without a family history of CVD, and those with hypertension or diabetes. All three forms of mortality correlated positively with advancing age and negatively with higher educational status, higher annual household income and greater physical activity (Table 2).

**Table 2:** Stratified analysis of mortality in COPD patients.

Characteristics	All-cause mortality (*n* = 2000)	Chi-square value	*p-*value	COPD-specific mortality (*n*_1_ = 665)	Chi-square value	*P-*value	CVD-specific mortality (*n*_2_ = 1,116)	Chi-square value	*P-*value
Sex		93.21	<0.0001		11.39	0.0007		18.32	<0.0001
Men	1,049(28.63)			328(8.95)			547(14.93)		
Women	951(19.66)			337(6.97)			569(11.76)		
Age (years)		1239.60	<0.0001		408.83	<0.0001		894.63	<0.0001
30-	19(2.99)			0(0.00)			4(0.63)		
40-	75(6.08)			13(1.05)			29(2.35)		
50-	415(14.66)			136(4.81)			172(6.08)		
60-	881(32.49)			307(11.32)			528(19.47)		
70–79	610(55.96)			209(19.17)			383(35.14)		
Marital status		175.29	<0.0001		51.37	<0.0001		145.14	<0.0001
Never married	37(42.05)			8(9.09)			21(23.86)		
Married	1,501(20.92)			498(6.94)			806(11.23)		
Sep/Div/Wid	462(37.35)			159(12.85)			289(23.36)		
Occupation		14.89	0.0006		4.57	0.1016		4.48	0.1062
Factory workers	7(9.21)			1(1.32)			4(5.26)		
Agricultural	1747(23.26)			590(7.85)			986(13.13)		
Other	246(26.94)			74(8.11)			126(13.80)		
Educational level		398.65	<0.0001		176.58	<0.0001		298.22	<0.0001
No formal school	804(36.53)			298(13.54)			498(22.63)		
Primary school	1,029(22.61)			327(7.18)			542(11.91)		
Junior high school or above	167(9.55)			40(2.29)			76(4.35)		
Annual household income (yuan)		83.53	<0.0001		43.86	<0.0001		72.65	<0.0001
<10,000	1,587(26.13)			548(9.02)			913(15.03)		
10,000–20,000	343(17.81)			99(5.14)			176(9.14)		
≥20,000	70(13.94)			18(3.59)			27(5.38)		
BMI (kg/m^2^)		260.00	<0.0001		219.25	<0.0001		149.71	<0.0001
<18.5	397(43.34)			180(19.65)			234(25.55)		
18.5–24.0	1,217(23.06)			385(7.30)			657(12.45)		
24.0–28.0	301(16.56)			75(4.13)			172(9.46)		
>28.0	85(17.35)			25(5.10)			53(10.82)		
Physical activity level		277.71	<0.0001		131.00	<0.0001		200.28	<0.0001
Low	1,415(30.40)			504(10.83)			827(17.77)		
Moderate	525(15.97)			146(4.44)			265(8.06)		
High	60(10.71)			15(2.68)			24(4.29)		
Fresh fruit intake frequency		61.15	<0.0001		29.41	<0.0001		36.76	<0.0001
Regular	220(15.49)			61(4.30)			116(8.17)		
Occasional	1780(25.14)			604(8.53)			1,000(14.12)		
Alcohol consumption status		217.17	<0.0001		151.26	<0.0001		132.34	<0.0001
Never regular	767(24.68)			288(9.27)			462(14.86)		
Ex-regular	231(40.88)			99(17.52)			135(23.89)		
Occasional	352(15.34)			115(5.01)			196(8.54)		
Monthly	28(19.58)			14(9.79)			21(14.69)		
Reduced intake	171(34.55)			62(12.53)			95(19.19)		
Weekly	451(23.80)			87(4.59)			207(10.92)		
CVD Family history		15.84	<0.0001		13.13	0.0002		10.15	0.0014
No	1790(24.24)			608(8.23)			1,003(13.58)		
Yes	210(18.82)			57(5.11)			113(10.13)		
Hypertension		102.09	<0.0001		4.16	0.0414		116.36	<0.0001
No	1743(22.19)			601(7.65)			942(11.99)		
Yes	257(39.72)			64(9.89)			174(26.89)		
Diabetes		31.09	<0.0001		2.61	0.1062		1.68	0.1954
No	1936(23.18)			648(7.76)			1,091(13.06)		
Yes	64(42.67)			17(11.33)			25(16.67)		

Higher mortality rates were seen in ex-smokers, those who had smoked for ≥50 years, who consumed a mean of <10 g cigarette equivalents/day, who smoked “other” tobacco products or reported mixed use, who inhaled to only oral depth and who commenced smoking at age < 15 years. A dose-dependent association was seen since higher baseline cumulative smoking exposure was linked to higher rates of all three forms of mortality (Table 3).

**Table 3:** Smoking habits and mortality in COPD patients.

Characteristics	All-cause mortality (*n* = 2000)	Chi-square value	*P*-value	COPD-specific mortality (*n*1 = 665)	Chi-square value	*P*-value	CVD-specific mortality (*n*2 = 1,116)	Chi-square value	*P*-value
Smoking category		299.94	<0.0001		178.16	<0.0001		143.39	<0.0001
Never smoked	580(15.77)			186(5.06)			329(8.95)		
Occasional smoker	167(23.62)			51(7.21)			93(13.15)		
Ex-smoker	379(40.45)			170(18.14)			213(22.73)		
Current smoker	874(27.49)			258(8.12)			481(15.13)		
Smoking duration (years)		497.57	<0.0001		154.07	<0.0001		283.25	<0.0001
Never smoked/occasional smoker	747(17.04)			237(5.40)			422(9.62)		
<30	217(18.17)			73(6.11)			117(9.80)		
30–50	694(30.51)			240(10.55)			370(16.26)		
>50	342(53.19)			115(17.88)			207(32.19)		
Cigarette equivalents/day (mean)		227.37	<0.0001		74.05	<0.0001		113.01	<0.0001
Never smoked/occasional smoker	747(17.04)			237(5.40)			422(9.62)		
<10	357(32.75)			115(10.55)			218(20.00)		
10–20	476(27.42)			174(10.02)			258(14.86)		
>20	420(32.56)			139(10.78)			218(16.90)		
Baseline cumulative smoking exposure (grams)		264.37	<0.0001		87.80	<0.0001		113.27	<0.0001
Never smoked/occasional smoker	747(17.04)			237(5.40)			422(9.62)		
1	283(26.80)			99(9.38)			170(16.10)		
100,000-	343(26.00)			116(8.79)			190(14.40)		
250,000-	627(36.01)			213(12.23)			334(19.18)		
Current smoking type		323.46	<0.0001		119.22	<0.0001		156.34	<0.0001
Never smoked/occasional smoker	747(17.04)			237(5.40)			422(9.62)		
Cigarettes	515(24.40)			173(8.20)			291(13.78)		
Other	675(38.55)			239(13.65)			376(21.47)		
Mixed tobacco use	63(24.80)			16(6.30)			27(10.63)		
Smoke inhalation depth		259.90	<0.0001		102.40	<0.0001		136.04	<0.0001
Never smoked/occasional smoker	747(17.04)			237(5.40)			422(9.62)		
Oral only	740(34.87)			267(12.58)			425(20.03)		
Deep inhalation	513(25.73)			161(8.07)			269(13.49)		
Smoking initiation age		229.77	<0.0001		85.48	<0.0001		120.48	<0.0001
Never smoked/occasional smoker	747(17.04)			237(5.40)			422(9.62)		
<15	172(38.39)			65(14.51)			107(23.88)		
15–25	751(29.50)			248(9.74)			397(15.59)		
≥25	330(29.41)			115(10.25)			190(16.93)		

Associations between smoking and mortality risk were analyzed using Cox proportional hazards models with adjustment for confounders. Compared with never smokers, ex-smokers had significantly increased risks of all-cause mortality (HR 1.56; 95% CI 1.36–1.80), COPD-specific mortality (HR 2.24; 95% CI 1.80–2.80), and CVD-specific mortality (HR 1.54; 95% CI 1.28–1.85). Current smokers showed a modest increase in all-cause mortality risk (HR 1.15; 95% CI 1.02–1.31) and CVD mortality risk (HR 1.20; 95% CI 1.01–1.42), while no significant association was observed for COPD-specific mortality. Occasional smokers did not show significant associations with mortality outcomes. For smoking duration, compared with never/occasional smokers, longer duration of smoking was associated with progressively increased risks of all-cause, COPD-specific, and CVD mortality. Participants who had smoked for >50 years had the highest risks of all-cause mortality (HR 1.40; 95% CI 1.20–1.62), COPD-specific mortality (HR 1.47; 95% CI 1.14–1.91), and CVD mortality (HR 1.44; 95% CI 1.18–1.75).

Consumption < 10 g tobacco/day was associated with increased risk of all cause, COPD-specific and CVD mortality relative to zero tobacco consumption and mortality risk rose with higher daily consumption. An intake of >20 g/day produced the greatest risk of increased COPD-specific (HR 1.66; 95% CI 1.27–2.16; 66% higher risk), all cause (HR 1.35; 95% CI 1.16–1.57; 35%) and CVD mortality (HR 1.34; 95% CI 1.10–1.65; 34%) relative to those who had never smoked or occasional smokers. Cumulative exposure progressively increased risk with ≥250,000 g/lifetime being associated with a 60% increase in COPD-specific (HR 1.60; 95% CI 1.26–2.04), 36% in all cause (HR 1.36; 95% CI 1.19–1.56) and 35% in CVD (HR 1.35; 95% CI 1.12–1.62) mortality.

An inverse relationship between mortality risk and age of smoking onset was found. Onset before the age of 15 years increased the risk of COPD-specific mortality by 71% (HR 1.71; 95% CI 1.28–2.29), CVD by 55% (HR 1.55; 95% CI 1.23–1.94) and all cause by 40% (HR 1.40, 95% CI 1.17–1.67; 40%) relative to those who had never smoked. By contrast, initiation after the age of 25 years raised mortality risk by 34% from COPD (HR 1.34; 95% CI 1.06–1.71), 22% from CVD (HR 1.22; 95% CI 1.01–1.47) and 16% from all causes (HR 1.16; 95% CI 1.01–1.33). Tobacco types and smoking methods affected mortality risk. “Other” tobacco products increased the risk of mortality from COPD by 71% (HR 1.71; 95% CI 1.35–2.16) and cigarettes increased risk by 33% (HR 1.33; 95% CI 1.09–1.64). “Other” tobacco products were also linked to 38% higher risk of all cause (HR 1.38; 95% CI 1.21–1.59) and 41% higher risk of CVD (HR 1.41; 95% CI 1.18–1.69) mortality compared with cigarettes (all-cause: HR 1.20; 95% CI 1.07–1.35, 20%; CVD: HR 1.26; 95% CI 1.08–1.48, 26%, Table 4).

**Table 4:** Smoking habits and disease-specific mortality risk in COPD patients: HRs (95%CIs).

Characteristics	All cause mortality		COPD-specific mortality		CVD-specific mortality	
	HR(95%CI)^a^	HR(95%CI)^b^	HR(95%CI)^a^	HR(95%CI)^b^	HR(95%CI)^a^	HR(95%CI)^b^
Smoking category						
Never smoked	1.00	1.00	1.00	1.00	1.00	1.00
Occasional smoker	1.07(0.90 ~ 1.27)	1.10(0.92 ~ 1.31)	1.00(0.73 ~ 1.37)	1.05(0.77 ~ 1.44)	1.01(0.80 ~ 1.28)	1.07(0.85 ~ 1.35)
Ex-smoker	1.57(1.37 ~ 1.80)	1.56(1.36 ~ 1.80)	2.19(1.76 ~ 2.74)	2.24(1.80 ~ 2.80)	1.50(1.25 ~ 1.80)	1.54(1.28 ~ 1.85)
Current smoker	1.17(1.03 ~ 1.32)	1.15(1.02 ~ 1.31)	1.12(0.90 ~ 1.40)	1.08(0.86 ~ 1.35)	1.18(1.00 ~ 1.38)	1.20(1.01 ~ 1.42)
Smoking duration (years)						
Never smoked/occasional smoker	1.00	1.00	1.00	1.00	1.00	1.00
<30	1.18(1.00 ~ 1.38)	1.20(1.02 ~ 1.40)	1.39(1.06 ~ 1.82)	1.47(1.12 ~ 1.94)	1.24(1.00 ~ 1.53)	1.31(1.06 ~ 1.62)
30–50	1.25(1.11 ~ 1.41)	1.24(1.10 ~ 1.40)	1.45(1.18 ~ 1.77)	1.42(1.15 ~ 1.74)	1.22(1.04 ~ 1.43)	1.23(1.05 ~ 1.45)
>50	1.45(1.25 ~ 1.68)	1.40(1.20 ~ 1.62)	1.61(1.25 ~ 2.08)	1.47(1.14 ~ 1.91)	1.47(1.22 ~ 1.78)	1.44(1.18 ~ 1.75)
Cigarette equivalents/day (mean)						
Never smoked/occasional smoker	1.00	1.00	1.00	1.00	1.00	1.00
<10	1.26(1.11 ~ 1.44)	1.24(1.09 ~ 1.42)	1.29(1.02 ~ 1.61)	1.27(1.01 ~ 1.60)	1.29(1.09 ~ 1.53)	1.31(1.11 ~ 1.55)
10–20	1.24(1.09 ~ 1.41)	1.23(1.08 ~ 1.41)	1.61(1.29 ~ 2.01)	1.57(1.25 ~ 1.97)	1.24(1.04 ~ 1.48)	1.25(1.05 ~ 1.50)
>20	1.35(1.17 ~ 1.57)	1.35(1.16 ~ 1.57)	1.67(1.29 ~ 2.17)	1.66(1.27 ~ 2.16)	1.32(1.08 ~ 1.61)	1.34(1.10 ~ 1.65)
Baseline cumulative smoking exposure (grams)						
Never smoked/occasional smoker	1.00	1.00	1.00	1.00	1.00	1.00
1	1.22(1.06 ~ 1.40)	1.20(1.04 ~ 1.38)	1.35(1.07 ~ 1.72)	1.35(1.06 ~ 1.72)	1.26(1.05 ~ 1.51)	1.28(1.06 ~ 1.53)
100,000-	1.23(1.07 ~ 1.42)	1.23(1.07 ~ 1.42)	1.43(1.13 ~ 1.81)	1.41(1.11 ~ 1.80)	1.25(1.04 ~ 1.50)	1.27(1.06 ~ 1.53)
250,000-	1.38(1.20 ~ 1.58)	1.36(1.19 ~ 1.56)	1.67(1.32 ~ 2.11)	1.60(1.26 ~ 2.04)	1.34(1.12 ~ 1.60)	1.35(1.12 ~ 1.62)
Current smoking type						
Never smoked/occasional smoker	1.00	1.00	1.00	1.00	1.00	1.00
Cigarettes	1.20(1.07 ~ 1.35)	1.20(1.07 ~ 1.35)	1.33(1.09 ~ 1.63)	1.33(1.09 ~ 1.64)	1.22(1.04 ~ 1.42)	1.26(1.08 ~ 1.48)
Other	1.44(1.26 ~ 1.64)	1.38(1.21 ~ 1.59)	1.84(1.46 ~ 2.31)	1.71(1.35 ~ 2.16)	1.46(1.22 ~ 1.74)	1.41(1.18 ~ 1.69)
Mixed tobacco use	1.13(0.87 ~ 1.48)	1.19(0.91 ~ 1.55)	1.05(0.62 ~ 1.77)	1.12(0.66 ~ 1.89)	0.93(0.62 ~ 1.39)	0.95(0.63 ~ 1.42)
Smoke inhalation depth						
Never smoked/occasional smoker	1.00	1.00	1.00	1.00	1.00	1.00
Oral only	1.33(1.17 ~ 1.50)	1.30(1.15 ~ 1.46)	1.66(1.35 ~ 2.03)	1.59(1.29 ~ 1.96)	1.38(1.18 ~ 1.62)	1.38(1.17 ~ 1.62)
Deep inhalation	1.22(1.08 ~ 1.38)	1.22(1.08 ~ 1.38)	1.29(1.05 ~ 1.60)	1.30(1.05 ~ 1.61)	1.18(1.00 ~ 1.39)	1.22(1.03 ~ 1.44)
Smoking initiation age						
Never smoked/occasional smoker	1.00	1.00	1.00	1.00	1.00	1.00
<15	1.44(1.21 ~ 1.72)	1.40(1.17 ~ 1.67)	1.81(1.36 ~ 2.42)	1.71(1.28 ~ 2.29)	1.58(1.26 ~ 1.97)	1.55(1.23 ~ 1.94)
15–25	1.31(1.16 ~ 1.47)	1.28(1.14 ~ 1.44)	1.47(1.20 ~ 1.80)	1.43(1.17 ~ 1.75)	1.27(1.09 ~ 1.49)	1.28(1.09 ~ 1.50)
≥25	1.14(0.99 ~ 1.31)	1.16(1.01 ~ 1.33)	1.32(1.04 ~ 1.67)	1.34(1.06 ~ 1.71)	1.17(0.97 ~ 1.40)	1.22(1.01 ~ 1.47)

^a^Adjusted for age and sex.

^b^Adjusted for age, sex, educational level, occupation, marital status, annual household income, alcohol consumption, BMI, MET (metabolic equivalent of task), fresh fruit intake, family history of cardiovascular disease, secondhand smoke exposure, hypertension, and diabetes.

### 3.2 Subgroup analysis

**Figure 1:**
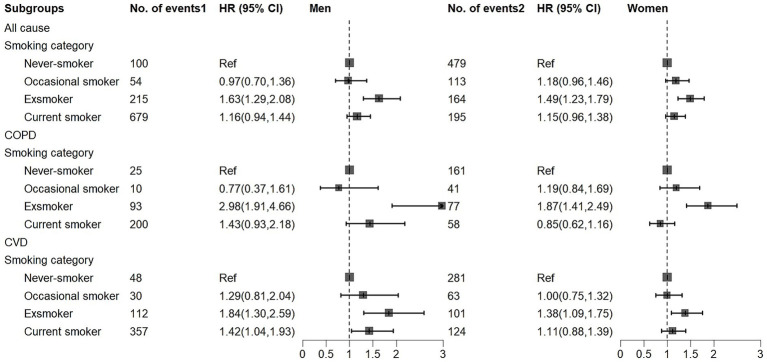
Adjusted hazard ratios (HRs) for all cause, COPD-specific, and CVD mortality stratified by smoking category in male and female subgroups. Adjusted risk ratios are plotted on floating absolute scales. The area of each black square is inversely proportional to the variance of the log risk ratio which also determines 95% confidence intervals (CIs).

Subgroup and sensitivity analyses were performed after adjustment of all Cox models. Ex-smokers showed significantly elevated risk of all cause, COPD-specific and CVD mortality in both sexes. Male current smokers had increased CVD mortality risk (HR 1.42; 95% CI 1.04–1.93, Figure 1).

**Figure 2:**
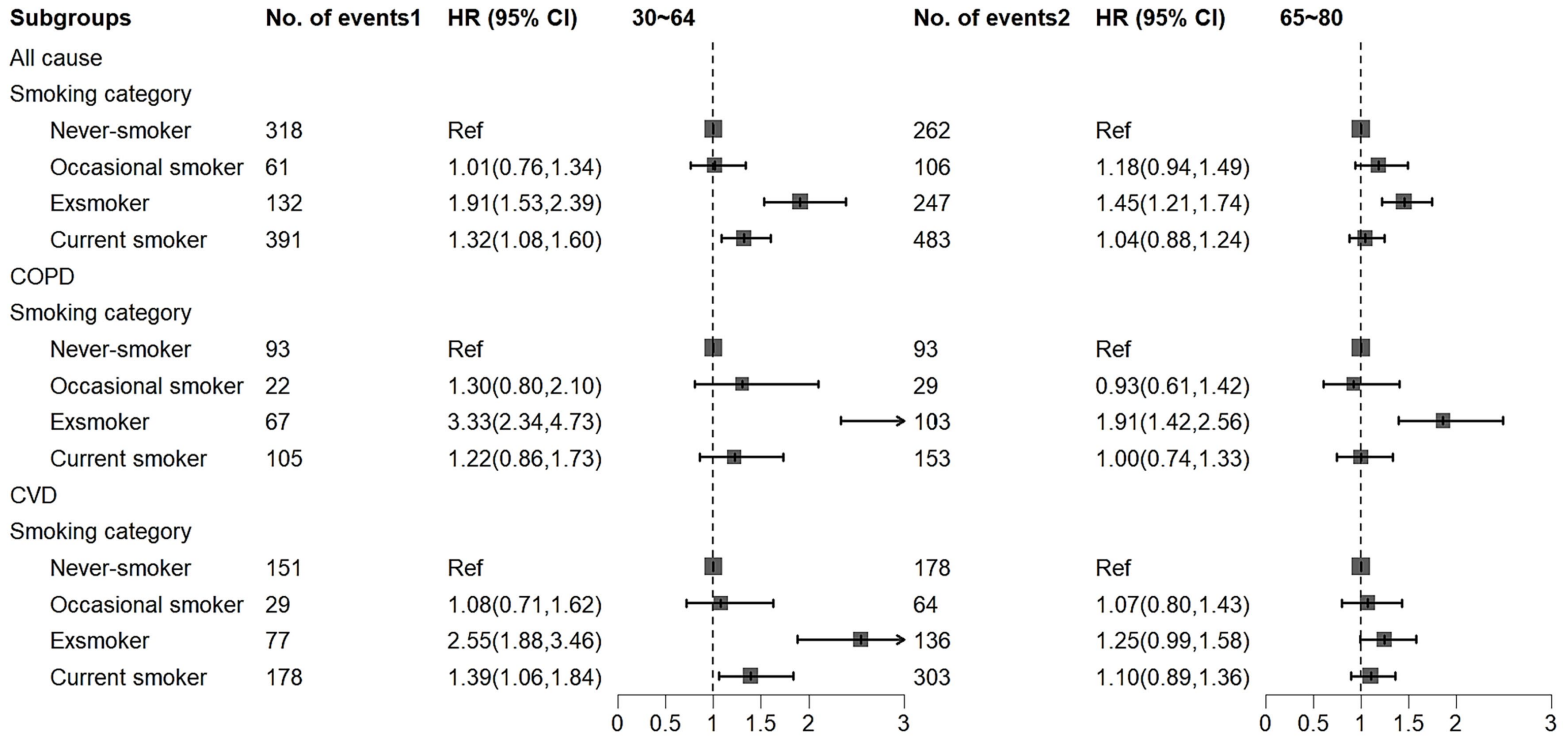
Adjusted hazard ratios (HRs) for all cause, COPD-specific, and CVD mortality, stratified by smoking category in 30–64 and 65–80 years age groups. Adjusted risk ratios are plotted on floating absolute scales. The area of each black square is inversely proportional to the variance of the log risk ratio which also determines the 95% confidence intervals (CIs).

Adjustment of the Cox proportional hazards model revealed elevated all cause and COPD mortality risk among ex-smokers in both the 30–64 and 65–80 year groups but elevated CVD mortality risk only in those aged 30–64 years (HR 2.55; 95% CI 1.88–3.36). Current smokers in the 30–64 years age group also showed higher all cause and CVD mortality risk but this was not present in the 65–80 grouping (Figure 2).

### 3.3 Sensitivity analysis

Sensitivity analyses, including exclusion of participants with respiratory symptoms, early deaths within 2 years of follow-up, and extreme values of cumulative smoking exposure, showed that associations between smoking category and mortality risk remained unchanged.

## 4 Discussion

The impact of smoking on mortality risk of COPD patients from Pengzhou, Sichuan, China was investigated through a prospective population-based cohort study. Adjustment for confounders showed smoking to be an independent risk factor for mortality from all causes, COPD and CVD. Significantly elevated risk of all three forms of mortality were present in ex-smokers, those who had smoked for >50 years, those who consumed > 20 g/day cigarette equivalents, users of “other” tobacco products, those reporting initial mixed tobacco use and oral-only inhalers. Baseline cumulative smoking exposure and age of smoking initiation allowed the demonstration of a dose–response relationship with mortality risk with higher cumulative exposure and younger commencement age giving progressively greater risk of all three mortality endpoints. Some of the current findings are consistent with previous reports. Elevated mortality risk among ex-smokers implies that smoking-induced lung damage may be irreversible in patients who have been diagnosed with COPD. However, the elevated risk observed among former smokers may also reflect reverse causation. Patients with more severe underlying disease or worsening health status are more likely to quit smoking, which may lead to an overestimation of mortality risk in this group. Therefore, the observed association does not necessarily indicate a harmful effect of smoking cessation, but rather may be influenced by baseline health status prior to quitting. GBD studies have associated smoking with negative, long-term cumulative effects which may not be completely reversed by late cessation (20). The finding that younger age of smoking initiation was associated with higher risk of all three mortality outcomes was similar to those of previous studies on the NHANES cohort. Early smoking initiation may impair lung development and increase biological susceptibility to chronic disease progression (21). In addition, the dose–response relationship between baseline cumulative smoking exposure and all three mortality outcomes was consistent with a Spanish cross-sectional study which showed that COPD risk increased with cumulative smoking exposure (22). Subgroup analyses showed heterogeneity by sex and age. Elevated risks among ex-smokers were observed in both sexes, while current smoking was associated with CVD mortality mainly in men. Most female participants were never smokers, suggesting that COPD in women may be more influenced by secondhand smoke, indoor air pollution, occupational exposures, and respiratory infections. By age, associations were stronger in participants aged 30–64 years but attenuated in those aged 65–80 years, particularly for current smoking and CVD mortality, possibly due to higher baseline mortality, competing risks, and survival bias.

The COPD patients using “other” tobacco products had significantly higher mortality risk than cigarette-only or mixed users. This aligns with the findings of Prescott et al., who suggested that non-cigarette tobacco products may impact pulmonary disease status in a manner dependent on inhalation patterns (23). In addition, a longitudinal US population study conducted by Christensen et al. found that cigar and pipe users were at substantially elevated COPD mortality risk compared to non-smokers (HR 7.66, 95% CI 6.09–9.64) (24) and Tasdighi et al. (25) similarly found that exclusive cigar or pipe use increased CVD mortality risk.

Previous studies have associated longer smoking duration with higher mortality risk among COPD patients and a similar pattern emerged from the current study in which those who had smoked for more than 50 years had the highest mortality risk. A clear duration-response relationship was seen. Indeed, previous studies have indicated that smoking duration may have a stronger link to adverse outcomes than smoking intensity, especially for patients with chronic diseases, such as COPD and lung cancer (26). Prolonged tobacco exposure may cause systemic oxidative stress and endothelial dysfunction which precipitates persistent airway inflammation, irreversible decline in lung function and increased mortality risk (27, 28).

By contrast with previous studies, a stable dose–response relationship between the number of cigarettes smoked per day and mortality risk was not observed in the current cohort. This inconsistency may be partly explained by post-diagnosis behavioral changes, including reduced cigarette consumption among patients with more severe symptoms. In addition, baseline daily smoking may not be representative of long-term exposure (29).

Daily smoking intensity showed a linear dose–response relationship with mortality and those who consumed >20 g/day had the highest risk of all three mortality outcomes. Greater intensity increases cumulative exposure to nicotine, tar and carbon monoxide, exacerbating airway inflammation, the decline in lung function and vascular endothelial damage (30). Previous major cohort studies, such as the British Doctors’ Study of Doll et al. (28) and Chen et al. (31), have reported significant positive correlations between smoking intensity and all-cause/cardiovascular mortality. The current results indicate the association of high-intensity smoking with especially pronounced risks in a COPD population. The observation that oral-only inhalers were at higher mortality risk than deep inhalers appears counterintuitive. Studies in Asian populations have shown shallow or non-inhaling smokers to be at significantly elevated risk of COPD. Studies in Asian populations have shown that non-inhaling smokers remain at increased risk of COPD mortality, suggesting that avoiding inhalation does not eliminate the harmful effects of tobacco exposure (32).

## 5 Conclusion

In summary, smoking significantly increased all-cause and cause-specific mortality risks among patients with COPD. The observed dose–response relationship, together with elevated risks associated with early smoking initiation and specific tobacco use patterns, highlights the importance of sustained smoking reduction and cessation interventions in this high-risk population. Notably, the persistently elevated risk among former smokers suggests that smoking cessation alone may not fully eliminate risk, underscoring the need for early intervention before substantial disease progression.

Future public health strategies should prioritize risk stratification among COPD patients according to smoking history, and implement targeted smoking cessation programs, particularly for individuals with long smoking duration or early smoking initiation. In addition, routine clinical management of COPD should incorporate individualized behavioral interventions combined with structured follow-up to prevent relapse and reduce long-term mortality risk.

### 5.1 Research advantages and limitations

We acknowledge several limitations. First, smoking behavior was self-reported at baseline, which may have introduced recall bias. Second, potential misclassification of COPD may exist due to the lack of bronchodilator use and incomplete spirometry measurements. Third, although multiple confounders were adjusted for, residual confounding cannot be excluded, particularly due to the lack of detailed clinical data on COPD severit. Finally, as most participants were from rural Sichuan, the generalizability of the findings to other populations requires further validation.

We believe our study to have some notable strengths. It was based on a large, representative rural cohort from the CKB project, with extensive exposure assessment, prolonged follow-up, and high-quality data collection. Unlike most previous studies in the general population, this study focuses on COPD patients, in whom the association between smoking and mortality may differ due to elevated baseline risk. In addition, by incorporating multiple dimensions of smoking exposure, including duration, intensity, cumulative exposure, and age at initiation, this study provides a comprehensive evaluation of dose–response relationships and cause-specific mortality.

## Data Availability

The raw data supporting the conclusions of this article will be made available by the authors, without undue reservation.
